# Varying Herbivore Population Structure Correlates with Lack of Local Adaptation in a Geographic Variable Plant-Herbivore Interaction

**DOI:** 10.1371/journal.pone.0029220

**Published:** 2011-12-29

**Authors:** Rodrigo Cogni, José R. Trigo, Douglas J. Futuyma

**Affiliations:** 1 Department of Ecology and Evolution, Stony Brook University, Stony Brook, New York, United States of America; 2 Departamento de Biologia Animal, Instituto de Biologia, Universidade Estadual de Campinas, Campinas, São Paulo, Brazil; University of Western Ontario, Canada

## Abstract

Local adaptation of parasites to their hosts due to coevolution is a central prediction of many theories in evolutionary biology. However, empirical studies looking for parasite local adaptation show great variation in outcomes, and the reasons for such variation are largely unknown. In a previous study, we showed adaptive differentiation in the arctiid moth *Utetheisa ornatrix* to its host plant, the pyrrolizidine alkaloid-bearing legume *Crotalaria pallida*, at the continental scale, but found no differentiation at the regional scale. In the present study, we sampled the same sites to investigate factors that may contribute to the lack of differentiation at the regional scale. We performed field observations that show that specialist and non-specialist polyphagous herbivore incidence varies among populations at both scales. With a series of common-garden experiments we show that some plant traits that may affect herbivory (pyrrolizidine alkaloids and extrafloral nectaries) vary at the regional scale, while other traits (trichomes and nitrogen content) just vary at the continental scale. These results, combined with our previous evidence for plant population differentiation based on larval performance on fresh fruits, suggest that *U. ornatrix* is subjected to divergent selection even at the regional scale. Finally, with a microsatellite study we investigated population structure of *U. ornatrix*. We found that population structure is not stable over time: we found population differentiation at the regional scale in the first year of sampling, but not in the second year. Unstable population structure of the herbivore is the most likely cause of the lack of regional adaptation.

## Introduction

Coevolution, the reciprocal evolutionary changes in interacting species driven by natural selection, has enhanced the diversity of life and has had profound effects on the structure of ecological communities [1]–[3]. Coevolution is a dynamic process that continually reshapes interactions among species across ecosystems, creating geographic mosaics over timescales sometimes as short as thousands or even hundreds of years [4]. In antagonistic interactions, coevolution may lead to constant shifts in the adaptive peaks that can result in local adaptation [5]–[6]. In many parasite-host interactions, such as herbivorous insects eating plants, the parasites are expected to exhibit more pronounced local adaptation than their hosts owing to their larger population sizes, shorter generation times and higher mutation rates [7]–[9]. This dynamic nature of local adaptation among coevolving species is an important mechanism in many theories within evolutionary biology, including evolution of ecological interactions, maintenance of genetic variation, maintenance of sexual reproduction, and the processes of parapatric and sympatric speciation [5].

Empirical studies of local adaptation in host-parasite interactions show highly variable outcomes. Many studies have detected local adaptation of parasites to their hosts, but others failed to detect it, or even found parasites to be locally maladapted [10], [11]. The possible reasons for this great variation in outcomes are largely unknown, and the great majority of studies do not investigate possible causal factors. Two recent meta-analysis studies failed to find many generalities on factors that may affect patterns of local adaptation in host-parasite systems [10], [11].

A key component to understand local adaptation is the spatial scale on which the coevolutionary processes occur [12]. Factors that affect the evolution of local adaptation may be scale-dependent. Therefore, studies that do not incorporate different geographical scales may miss coevolutionary dynamics that occur at either larger or smaller scales [3]. The greater the distance among the populations, the larger the expected difference in the traits related to the ecological interaction [13]–[15], because distant sites are likely to differ more in both abiotic and biotic factors [16], and because gene flow among populations also depends on the geographical scale [17].

In a previous study, we investigated local adaptation of the seed predator arctiid moth *Utetheisa ornatrix* to its host plant, the pyrrolizidine alkaloid-bearing legume *Crotalaria pallida* [18]. Herbivore fitness was measured as larval performance on unripe seeds from different plant populations in a common-garden to test for genetic differences among populations. In a search for “regional adaptation” using three populations from Southeast Brazil (each population ca. of 0.2 Km^2^ in area and *ca.* 150 Km apart for each other), we did not find evidences of adaptive differentiation of the herbivore, although we did find a statistically significant interaction between herbivore sex and plant population. This interaction indicates that the plant populations were differentiated at the regional scale. In a “continental scale” comparison of populations from Brazil and Florida, the herbivore showed adaptation to its host plant; for both moth populations the pupae were heavier when the larvae ate plants from the sympatric than plants from the allopatric host. These results, showing that adaptation by the herbivore can evolve, but nevertheless has not evolved at the regional scale, impelled us to investigate possible causes for the lack of regional adaptation.

In the present study, we sampled the same sites to investigate factors that may contribute to the lack of differentiation at the regional scale. First, we made field observations that found that specialist and non-specialist polyphagous herbivore damage varies among populations at both scales. Second, with a series of common-garden experiments we showed that some plant resistance traits vary at the regional scale. These results, combined with our previous evidence for plant population differentiation based on larval performance on unripe seeds, suggest that *U. ornatrix* is subjected to divergent selection at the regional scale. These factors indicate that regional adaptation is expected to evolve. Finally, with a microsatellite study we showed that population structure of *U. ornatrix* is not stable over time. This unstable population structure of the herbivore is the most likely cause for the lack of adaptation at the regional scale.

## Methods

### Study system and plant resistance traits

*Crotalaria pallida* is an annual plant native to Africa and currently occurs at high densities from southern Brazil to the southeastern United States. There is no clear evidence about the New World introduction; possibly it was transported from Africa during the slave trade in the sixteenth century [19]. *C. pallida* is self-compatible and sets fruits autogamously [20], but is also bee-pollinated. *C. pallida* lacks any mechanism for long-distance seed dispersal. In the neotropics, *Utetheisa ornatrix* is one of the main natural enemy of *Crotalaria* plants; the generalist pod-borer *Etiella zinckenella* (Lepidoptera: Pyralidae) was also found as an important herbivore in some localities (Trigo pers. obs.). *U. ornatrix* originally fed on native *Crotalaria* species, but currently uses *C. pallida* as its host in several locations [21]–[24]. Because of its high abundance, *C. pallida* is the main host of *U. ornatrix* in several locations [21]–[24]. By preying on the seeds, *U. ornatrix* can have a significant impact on the fitness of *Crotalaria* plants; up to 20% of *C. pallida* fruits in the field may be damaged by *U. ornatrix* [21], [25].

Plants employ an enormous diversity of chemical, mechanical and biotic resistance traits to avoid herbivores and pathogens [26]. Research in the past decades has been searching for single silver bullet traits, but, for the vast majority of plant-herbivore systems, it has been difficult to determine which trait is the most important for a particular herbivore [27]. From all the possible traits in *Crotalaria* that may affect herbivory, we measured four traits based on the possible relevance to *U. ornatrix* and the existence of well-established protocols. We investigated pyrrolizidine alkaloids (PAs), extrafloral nectaries (EFNs), carbon and nitrogen content, and trichome density. These represent a small subset of all the possible traits that may affect *U. ornatrix*. For example, there is sparse information that *Crotalaria* plants may also have other chemical defenses such as isoflavonoids, non-protein amino acids and proteinase inhibitors [28]–[32], classes of compounds that we were not equipped to measure.

The constitutive presence of PAs is considered to be the major resistance trait in *Crotalaria* plants [31]. PAs encompass a group of about 360 chemical structures with occurs in a restricted number of higher plant clades [33]. PAs have deterrent and toxic effects on a variety of non-specialist polyphagous herbivores [34]–[35]. However, *U. ornatrix* larvae are able to sequester PAs from *Crotalaria* host plant. The PAs not only protect larvae and adults, but are also transmitted from the female (and from males through nuptial gift) to eggs. Males also modify the PAs into a courtship pheromone [22], [36], [37]. Because PAs are beneficial, plants with higher PA concentration may be more rather than less desirable to *U. ornatrix* [24].

*C. pallida* has EFNs located on the base of the peduncle that remain active from the early development of flowers to formation of mature fruits. EFNs are sugar-producing plant structures not directly related to pollination. EFNs attract ants that exhibit aggressive behavior towards herbivores [38]. In *C. pallida*, ants attracted to EFNs frequently patrol the fruit pods, expelling *U. ornatrix* larvae that are outside the fruit [25], [39]; predaceous wasps are also attracted to EFNs and prey upon *U. ornatrix* larvae (Trigo pers. obs.).

We also investigated two other plant traits: carbon and nitrogen content of the seeds and trichome density on the leaves. Carbon and nitrogen content are not considered a resistance trait “per se”, but relative nitrogen content is important in nutritional quality for herbivores, which generally prefer plants with higher nitrogen content [40], [41]. Leaf trichomes affect leaf herbivores [42], and may affect *U. ornatrix* neonate larvae, which eat leaves before entering the fruit to prey on the seeds.

### Populations studied

**Figure 1:**
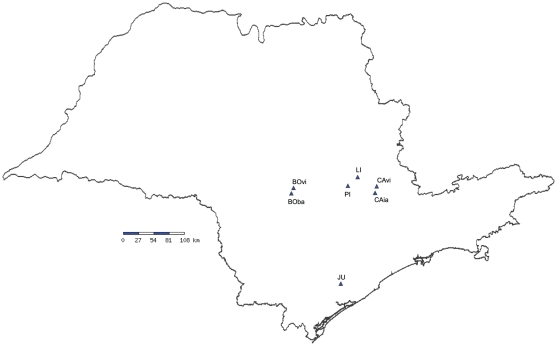
Map of populations included in this study from São Paulo State, SE Brazil.

In May 2005, we collected *C. pallida* seeds and moths from three sites in São Paulo State, Southeast Brazil (Table 1; Figure 1). In April 2006, we collected at Archbold Biological Station in central Florida, US (Table 1). These are the same sites used in our previous study of local adaptation [18]. In each site we collected seeds from at least 30 individual plants for the common garden studies and 16 adult moths for the microsatellite study (Table 1). In all these sites *C. pallida* was the predominant host; in some sites other *Crotalaria* species (*C. incana*, *C. lanceolata*) were present at a very low density (less than 5%). The original host plant from each adult moth collected could not be determined, but since *C. pallida* is the predominant host in all sites, we can assume that almost all moths collected originate from this species. Field studies for the quantification of herbivore incidence were carried out in January 2007 in the Brazilian sites, and in November 2009 for the site in Florida. In April 2008 we performed additional collections in Brazil for the microsatellite study; we collected a larger number of individuals (22–29 moths) in the sites previously studied (except the JU05 population, which had been destroyed by fire), and we also collected from four additional sites (Table 1). In two of these sites moths were collected from an alternative host plant, *Crotalaria trichotoma* (Table 1). All necessary permits were obtained for the described field studies. Permits to collect moths and plants in all sites in Brazil were provided by Instituto Brasileiro do Meio Ambiente e dos Recursos Naturais Renováveis (IBAMA) (permit numbers: 100/05- CGFAU/LIC and 027/05 – COMON). None of the sites were privately-owned or protected in any way and the studies did not involve endangered or protected species. The permission to perform field studies and collection of moths and plants at Archbold Biological Station in Florida, US, was provided by Dr. Mark Deyrup. The field studies did not involve endangered or protected species. The permit to export live organisms from Brazil to the US was provided by Instituto Brasileiro do Meio Ambiente e dos Recursos Naturais Renováveis (IBAMA) (permit number 08BR001899/DF and 0126797 BR). The permit to import live organisms from Brazil to the US was provided by the United States Department of Agriculture (USDA) Animal and Plant Health Service (APHIS) (permit number 71956).

**Table 1:** Populations used in the study.

Locality	Year collected	Coordinates	Altitude (m)	Acronym	# individuals *U. ornatrix*
Campinas (Village)^1^	2005	22°45′12″S 47°03′20″W	605	CAvi05	16
Campinas (Village)	2008	22°45′12″S 47°03′20″W	605	CAvi08	27
Campinas (IAC)^2^	2008	22°51′21″S 47°04′27″W	629	CAia08	25
Botucatu (Vitoriana)^1^	2005	22°46′45″S 48°24′14″W	540	BOvi05	16
Botucatu (Vitoriana)	2008	22°46′45″S 48°24′14″W	540	BOvi08	29
Botucatu (Bairro)	2008	22°52′07″S 48°26′24″W	785	BOba08	23
Juquiá^1^	2005	24°19′55″S 47°38′15″W	21	JU05	16
Limeira	2008	22°36′18″S 47°21′45″W	569	LI08	24
Piracicaba^2^	2008	22°44′29″S 47°31′17″W	518	PI08	22
Florida (Lake Placid)^1^	2006	27°11′13″N 81°20′19″W	30	FL06	16

1 Populations used on the plant resistance study.

2 *Utethesia ornatrix* collect on the alternative host *Crotalaria trichotoma*.

### Herbivore incidence in the field

We evaluated herbivore presence and damage in the field at a single point in time. Selection pressure in the wild is known to vary seasonally and among year; therefore the precise estimation of selection pressure requires several years of field studies and is beyond the scope of this study. Since *C. pallida* fruits stay on the plant for several weeks before autochoric dispersal, our measurement reflects the herbivore attacks that happened during the few weeks before sampling. In each site we examined 30 plants that were at least 5 meters from each other. The herbivore species found were the same as those that were previously identified in the system [21]. For each plant we collected all fruits, opened them in the laboratory, and recorded the proportion of pods that were attacked by the two most common herbivores - *U. ornatrix* and *E. zinckenella*. We avoided plants that had already started seed dispersal and plants with only young fruits, to restrict sampling to plants that had been exposed to herbivores at approximately the same time. Pods attacked by *U. ornatrix* can be easily identified by the characteristic opening that the larva makes to enter the pod, while pods attacked by *E. zinckenella* do not have an opening [21], [43]. The proportion of pods attacked per plant was compared among the sites by a Kruskal-Wallis test because the data were not normally distributed even after transformation.

### Plant resistance traits

We investigated genetic differences among sites in plant resistance traits with a series of common-garden experiments.

#### Common environment conditions

We grew plants using seeds from different sites in a common environment as previously described [18]. In the 2005 experiment (regional comparison) we used the greenhouse of the Institute of Biology at State University of Campinas in São Paulo State, Brazil. In the 2006 experiment (continental comparison) we used the greenhouse at the Life Science Building at Stony Brook University in Stony Brook, NY, US. In 2005, we started the plants in May and collected the samples for chemical analyses and trichome counts in November. In 2006, we started the plants in April and collected the samples in October. Although the conditions of the greenhouses differed between the countries, we have evidence showing that any such difference did not affect our results [18]. For the EFNs experiment, we used a set of plants grown in the Brazilian greenhouse in 2005. The plants were started in May and the experiment was carried out in October (see below). For the EFNs experiment comparing Brazil and Florida, we grew plants in 2008 from the same set of seeds used in the 2006 experiments. The plants were grown under the same conditions, except that they were grown outside, in the city of Mogi Mirim (SP, southeastern Brazil). We did not observe any herbivory on the plants grown outside that could have influenced the results. The plants were started in August 2008 and the experiments carried out in February and March 2009.

Our common garden experiments were designed to test genetic differences, but since plants were grown from seeds collected in the field, we cannot completely exclude the hypothesis that observed differences were due to maternal effects. We addressed this issue by growing a second generation of plants from the 2006 experiment in 2007 in Stony Brook and measuring trichome densities in 30 individuals per site. For both sites, trichome density did not differ between the first and the second generations (CAvi05: t = 0.128, df = 96, p = 0.90; FL06: t = 0.212, df = 97, p = 0.829) indicating that the differences observed were not caused by maternal effects.

#### Concentration of PAs

We collected approximately 10 unripe fruits per individual common-garden plant in 50 ml EtOH. All the fruits were at the same developmental stage as offered to larvae in our local adaptation study [18]. The samples (15–16 per site) were triturated and total PAs were extracted and quantified by the colorimetric method [44]. For each individual, the extract was divided in four parts and four replicate readings were performed. We used Dixon's Q-test to detect possible outliers among the four replicated spectrophotometer readings. A calibration curve was constructed using pure retrorsine extracted from the inflorescences of *Senecio brasiliensis* (Asteraceae) [44]. We initially collected and analyzed the entire unripe fruit, but because *U. ornatrix* larva eats only the unripe seeds, we later analyzed only seeds. We constructed a correction curve to estimate the PA concentration in the seeds from the PA concentration in the entire fruit. The curve was constructed from 20 samples in which we separated the green seeds from the other parts of the fruit before quantifying the PAs as described above. Therefore, the concentration of PAs in the seeds was estimated as y = 2.1105x−0.0003 (R^2^ = 0.934), where x is the total concentration of PAs in the entire fruit. GC/MS analyses [45] confirmed that all the sites studied have a similar proportion of two PAs in the unripe seeds: intergerrimine (*ca.* 15%) and usaramine (*ca.* 85%).

#### Carbon and Nitrogen content

Fruits were collected and dried at 60°C for 72 hours (13–16 individuals per site). The seeds were separated and ground to a fine powder with liquid nitrogen and further dried at 60°C. About 6 mg per sample were analyzed in a CHN elemental analyzer.

#### Trichome density

We estimated trichome density on the lower surface of leaves. Thirty individuals per site were sampled at the regional scale. At the continental scale 68 individuals were sampled from the Brazilian site and 69 from the Florida site. For each individual the central leaflet of the third fully developed leaf from the shoot tip was collected. A leaf-disc was cut at a central position and, by using a compound microscope, the number of trichomes was counted in 1 mm^2^ using a 10×10 mm grid. For each leaf-disc nine 1 mm^2^ squares were counted, avoiding squares with major veins. The nine counts were then averaged for an estimate of the number of trichomes.

#### Extrafloral nectaries (EFNs)

For the regional comparison in 2005, 42 plants from each of the three sites were transferred from the greenhouse to the borders of the Santa Genebra conservation unit (in Campinas – SP). One plant from each site was placed in each block (42 blocks of 3 plants each). All plants were in the reproductive stage (flowers or young fruits), and plants in the same block had similar size and phenological stage. Blocks were 10 meters apart. Ten days after the plants were transferred we checked for the presence of ants visiting the EFNs and counted the total number of ants per plant during a 30 second interval. To estimate ant aggressiveness, a termite worker (*Nasutitermes* sp.) was glued on a reproductive stem of each plant and observed for 10 minutes to see if it was attacked by ants (a standard procedure in ant-plant studies [46], [47]). The ants visiting the EFNs during the experiment were *Camponotus* sp., *Brachymyrmex* sp., *Pheidole* sp. and *Crematogaster* sp. For the continental comparison, 25 plants from each site were transferred to a grass field at the Mogi Mirim City Zoo (Mogi Mirim – SP). The same procedures described above were used. The observed ants visiting the EFNs were *Brachymyrmex* sp., *Camponotus* (3 species), *Crematogaster* sp., *Pheidole* (2 species) and *Pseudomyrmex* sp. The ant assemblages visiting the EFNs are typical species in neotropical EFN-bearing plants [48], [49]. The two localities where these experiments were carried out, did not have naturally grown *Crotalaria* plants, and were near the site where the Ca population was collected.

#### Statistical analyses

At the regional scale, differences among the three origin sites for each plant resistance trait were tested with one-factor ANOVAs. At the continental scale differences between the two sites were tested with t-tests. Concentration of PAs and trichome density variables were log transformed to obtain normal distribution. For the EFNs experiments in 2005, the number of ants visiting plants from each site was compared by a Kruskal-Wallis test because the data was not normally distributed even after transformation. For the 2009 EFNs experiment, the mean number of visiting ants for each original site was compared by a paired t-test. The percent of termites attacked by ants was compared by χ^2^ tests.

### Herbivore genetic structure - Microsatellites study

To investigate how the population structure of the herbivore may affect the patterns of local adaptation we performed a microsatellite study with samples collected in two different years.

#### DNA extraction and microsatellite amplification

Moth genomic DNA was extracted with Qiagen DNeasy tissue kit; adult moths preserved in EtOH were ground in liquid nitrogen after the removal of wings and the abdomen. Five microsatellite loci developed by Bezzerides *et al.* [50] were amplified (Table S1). PCR conditions were similar to Bezzerides *et al.* [50], with the exception that GoTaq polymerase and dNTPs from Promega were used and the final volume of the reactions was 25 µL. Amplifications that failed on individual samples were repeated one time. Fragments were analyzed with an ABI3730 DNA Analyzer with the size standard LIZ 500 (Applied Biosystems). Allele sizes were estimated using GENEMAPPER 3.0 (Applied Biosystems) and verified by manual scoring.

#### Data analyses

We used GENEPOP (vers. 4.0) [51] to test loci for linkage disequilibrium (1,000 dememorisations; 10,000 batches; 10,000 iterations per batch). None of the loci were in linkage disequilibrium (only one of the 100 tests within sites was individually significant at α = 0.05). Pairs of loci tested across all sites were not significant either (Table S2). To contrast genetic diversity within the sites we used GENEPOP to calculate number of alleles, and the expected and observed number of heterozygotes for each locus. We used GENEPOP to test for deviations from Hardy-Weinberg equilibrium (exact test; 1,000 dememorisations; 100 batches; 1,000 iterations per batch) with the sequential Bonferroni correction for multiple testing [52]. Pairwise population differentiation was tested with exact G tests in GENEPOP (genic differentiation) for each locus and across all loci (assuming statistical independence across loci). This procedure tests the null hypothesis that alleles are drawn from the same distribution in the different sites. Pairwise F_st_ values were calculated in GENEPOP by the “weighted” analysis of variance for each locus and across all loci. This method uses ANOVA mean sum of squares (for gametes, individuals and populations) to estimate F statistics [53], [54]. The estimation across all loci is a modification of the method using a weighted sum of each locus [54], [55] that gives higher weight to loci with larger sample sizes [51].

## Results

### Geographical differences in herbivore pressure

Only three species of herbivores were observed feeding on *C. pallida* seeds: the specialist *U. ornatrix*, and two non-specialist polyphagous insects, *E. zinckenella*, and the red-shouldered stink bug *Thyanta perditor* (Hemiptera: Pentatomidae), that feeds outside the fruit, unlikely the other two herbivores that feed inside the fruit. The same species were observed in all sites sampled, except for the stink bug, which was not observed in Florida. The proportion of pods attacked by the two most common herbivores differed among the sites (Table 2; attacks by *U. ornatrix*: H = 37.2, p<0.0001, N = 120; attacks by *E. zinckenella*: H = 22.22, p<0.0001, N = 120). There were differences in herbivore incidence even at the regional scale. Even when only the three Brazilian sites were compared the differences in the proportion of pods attacked were significant. A larger proportion of pods was attacked by *U. ornatrix* in the CAvi site (Table 2; H = 14.644; p = 0.001; N = 90). The proportion of pods attacked by *E. zinckenella* was higher in the BOvi site (Table 2; H = 11.687; p = 0.003; N = 90).

**Table 2:** Field differences among populations in the proportion of pods attacked per plant by *Utetheisa ornatrix* and *Etiella zinckenella*.

	Population			
	SE Brazil			US
	BOvi	CAvi	JU	FL
*Utetheisa ornatrix*	0.028±0.009	0.132±0.040	0.015±0.005	0.189±0.036
*Etiella zinckenella*	0.081±0.018	0.020±0.008	0.022±0.008	0.094±0.018

Populations are from São Paulo State, Southeastern Brazil, and Central Florida, US. Values are mean ± SE.

### Plant resistance traits

**Figure 2:**
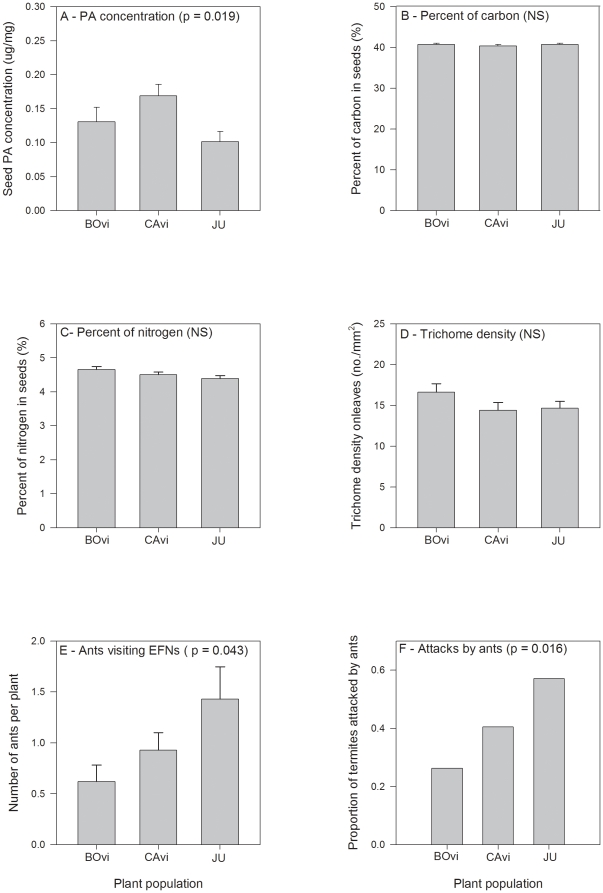
Among population variation on *Crotalaria pallida* resistance traits at a regional scale. (A) pyrrolizidine alkaloids (PAs) in unripe seeds, (B) carbon content of unripe seeds, (C) nitrogen content of unripe seeds, (D) trichome density on leaves, (E) number of ants attracted to extrafloral nectaries (EFNs), and (F) percent of termites bites attacked by ants. Values are mean + SE (A–E). Plants were grown in a common greenhouse environment and are from three localities in São Paulo State, SE Brazil: BO = Botucatu, CA = Campinas and JU = Juquiá. P values and ns (non-significant) indicate the effect of population on ANOVA tests (A–D), Kruskal-Wallis (E) or χ^2^ test (F).

At the local scale, the three sites from Brazil differed in the PAs' concentration in the seeds (Figure 2A; Table 3A). There were no differences among the sites in the seeds' carbon and nitrogen content, or in the trichome density on leaves (Figures 2 B–D; Table 3B–D). Ant attractiveness to EFNs also differed among the sites; there was a slightly higher number of ants visiting the plants from JU (H = 6.305; p = 0.043; N = 126) and the percent of termite baits attacked by ants differed (χ^2^ = 8.32; d.f. = 2, p = 0.016) (Figures 2 E–F).

**Table 3:** Effect of population origin on *Crotalaria pallida* resistance traits at a regional scale.

Source	d.f.	Mean squares	F-ratio	P
**(A) PAs concentration** *				
Population	2	1.310	4.32	0.019
Error	43	0.303		
**(B) Carbon content**				
Population	2	0.578	1.246	0.298
Error	43	0.464		
**(C) Nitrogen content**				
Population	2	0.278	2.378	0.105
Error	43	0.117		
**(D) Trichome density**				
Population	2	0.163	1.594	0.209
Error	87	0.102		

(A) pyrrolizidine alkaloids (PAs) in green seeds, (B) carbon content of green seeds, (C) nitrogen content of green seeds, and (D) trichome density on leaves. Plants were grown in a common greenhouse environment and are from three localities in São Paulo State, SE Brazil.

*indicates significant differences.

**Figure 3:**
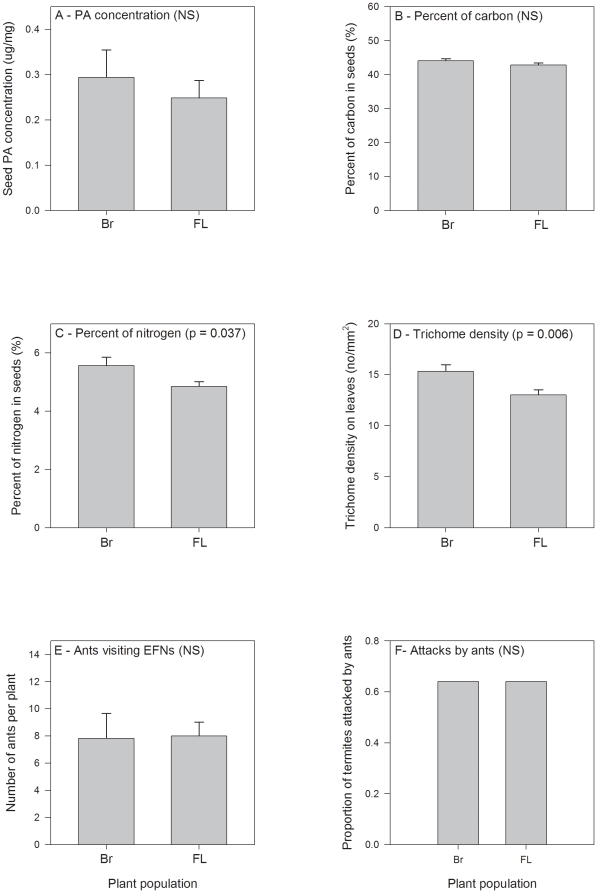
Population variation of *Crotalaria pallida* resistance traits at a continental scale. (A) pyrrolizidine alkaloids (PAs) on unripe seeds, (B) carbon content of unripe seeds, (C) nitrogen content of unripe seeds, (D) trichome density on leaves, (E) number of ants attracted to extrafloral nectaries (EFNs), and (F) percent of termites bites attacked by ants. Values are mean + SE (A–E). Plants were grown in a common environment and are from Campinas in São Paulo State in SE Brazil (Br) and Central Florida (Fl) in the US. P values and ns (non-significant) indicate between population differences on t tests (A–E) or χ^2^ test (F).

At the continental scale, there was no difference in the PAs' concentration in seeds between Florida and the Brazilian site (Figure 3A; t = 0.213, d.f. = 30, p = 0.833). There was no difference between the populations in the seeds' carbon content (Figure 3B; t = 1.383, d.f. = 27, p = 0.178). On the other hand, the seeds from Brazil have higher nitrogen content (Figure 3C; t = 2.18, d.f. = 28, p = 0.037), and more trichomes than plants from Florida (Figure 3D; t = 2.82, d.f. = 135, p = 0.006). The average number of ants per plant (t = 0.099, d.f. = 24, p = 0.922) and the percent of termite baits attacked by ants (χ^2^ = 0.00; d.f. = 2, p>0.99) did not differ (Figures 3 E–F).

### Herbivore genetic structure

Genetic diversity for each locus on each site is described in Table S3. Locus Utor 2 deviated from Hardy-Weinberg equilibrium in all sites (Table S3), suggesting the presence of null alleles. The three sites collected in 2005 showed significant differentiation (Table 4). Surprisingly, the sites collected in 2008 were not significantly differentiated (Table 5). For the two sites sampled in both 2005 and 2008, there was significant pairwise differentiation in 2005 and no differentiation in 2008. The test of 2005 *vs.* 2008 for each of these two localities revealed significant temporal differentiation (Table 6). The host-plant present in each site did not influence the genetic structure of *U. ornatrix*. The two sites with the alternative host *C. trichotoma*, were not differentiated from sites where only the most common host, *C. pallida*, was present (Table 5).

**Table 4:** *Utetheisa ornatrix* population differentiation at the regional scale in 2005.

*Utor 2*			
	BOvi05	CAvi05	JU05
**BOvi05**	-	*<0.001*	0.079
**CAvi05**	0.031	-	*0.01*
**JU05**	−0.030	0.012	-

*Utor7*			
	BOvi05	CAvi05	JU05
**BOvi05**	-	*<0.001*	*0.008*
**CAvi05**	0.068	-	*0.01*
**JU05**	0.080	0.028	-

*Utor28*			
	BOvi05	CAvi05	JU05
**BOvi05**	-	0.06	*0.004*
**CAvi05**	0.035	-	*<0.0001*
**JU05**	0.110	0.165	-

*UtorTAC*			
	BOvi05	CAvi05	JU05
**BOvi05**	-	*0.009*	*<0.0001*
**CAvi05**	0.057	-	*<0.001*
**JU05**	0.101	0.037	-

*Across all loci*			
	BOvi05	CAvi05	JU05
**BOvi05**	-	*<0.0001*	*<0.0001*
**CAvi05**	0.050	-	*<0.0001*
**JU05**	0.060	0.049	-

Above diagonal: p value for genic differentiation (exact G test) for each population pair, for each locus and across all loci (significant values in italic). Below diagonal: pairwise F_st_ values. Locus Utor 10 was not included because it did not amplify in any individual of BOvi05 and CAvi05 populations.

**Table 5:** *Utetheisa ornatrix* population differentiation at the regional scale in 2008.

*Utor2*						
	BOba08	BOvi08	CAia08	CAvi08	LI08	PI08
**BOba08**	-	0.668	0.144	0.348	0.169	*0.*304
**BOvi08**	−0.008	-	0.145	0.245	0.*009*	0.429
**CAia08**	−0.002	0.000	-	0.069	0.080	0.359
**CAvi08**	−0.015	0.009	−0.008	-	0.515	0.263
**LI08**	−0.013	0.029	0.001	−0.020	-	*0.*060
**PI08**	−0.010	−0.014	−0.006	0.007	0.018	-

*Utor7*						
	BOba08	BOvi08	CAia08	CAvi08	LI08	PI08
**BOba08**	-	0.080	0.471	0.209	0.648	*0.*937
**BOvi08**	0.005	-	0.166	0.927	0.736	0.710
**CAia08**	−0.007	−0.015	-	0.107	0.736	0.815
**CAvi08**	0.012	−0.016	−0.008	-	0.657	0.897
**LI08**	−0.012	−0.022	−0.022	−0.011	-	*0.*962
**PI08**	−0.019	−0.019	−0.022	−0.018	−0.026	-

*Utor10*						
	BOba08	BOvi08	CAia08	CAvi08	LI08	PI08
**BOba08**	-	0.180	0.259	0.230	0.*026*	*0.017*
**BOvi08**	0.020	-	1.00	0.297	0.215	0.622
**CAia08**	−0.040	−0.154	-	0.375	0.121	0.536
**CAvi08**	0.030	−0.045	−0.058	-	0.779	0.*028*
**LI08**	0.118	−0.041	−0.016	−0.082	-	*0.012*
**PI08**	0.155	−0.117	−0.130	−0.005	0.052	-

*Utor28*						
	BOba08	BOvi08	CAia08	CAvi08	LI08	PI08
**BOba08**	-	0.493	0.255	0.399	0.*05*	*0.012*
**BOvi08**	−0.011	-	0.655	0.872	0.517	0.*038*
**CAia08**	−0.010	−0.003	-	0.810	0.248	0.287
**CAvi08**	−0.006	−0.016	0.001	-	0.796	0.317
**LI08**	−0.003	−0.016	−0.004	−0.019	-	*0.027*
**PI08**	0.001	0.001	−0.007	−0.014	−0.006	-

*UtorTAC*						
	BOba08	BOvi08	CAia08	CAvi08	LI08	PI08
**BOba08**	-	0.555	0.687	0.357	0.762	*0.*128
**BOvi08**	0.004	-	0.448	0.234	0.395	0.197
**CAia08**	0.006	−0.010	-	0.537	0.607	0.330
**CAvi08**	0.026	0.004	−0.008	-	0.650	0.894
**LI08**	−0.008	−0.006	−0.014	−0.008	-	*0.*088
**PI08**	0.037	0.001	−0.012	−0.018	−0.001	-

*Across all loci*						
	BOba08	BOvi08	CAia08	CAvi08	LI08	PI08
**BOba08**	-	0.294	0.315	0.280	0.051	*0.008*
**BOvi08**	0.000	-	0.449	0.573	0.089	0.216
**CAia08**	−0.004	−0.014	-	0.201	0.188	0.588
**CAvi08**	0.007	−0.005	−0.009	-	0.948	0.251
**LI08**	0.000	−0.004	−0.018	−0.019	-	*0.003*
**PI08**	0.012	−0.015	−0.017	−0.005	0.001	-

Above diagonal: p value for genic differentiation (exact G test) for each population pair, for each locus and across all loci (significant values in italic). Below diagonal: pairwise F_st_ values.

**Table 6:** Temporal differentiation in population structure of *Utetheisa ornatrix*.

Locality		Utor2	Utor7	Utor28	UtorTAC	Across all loci
**BOvi**	p value (G test)	*0.001*	*0.003*	0.484	0.117	<0.*0001*
	F_st_	0.036	0.079	−0.008	0.0003	0.032
**CAvi**	p value (G test)	*0.007*	*0.018*	0.550	0.465	0.*008*
	F_st_	0.030	0.021	−0.002	0.016	0.021

Two localities (BOvi and CAvi) were sampled in 2005 and 2008, and differentiation between years was tested with exact G tests for genic frequencies. F_st_ values were calculated for each locality between the years for each locus and across all loci. Locus Utor 10 was not included because it did not amplify in any individual of BOvi05 and CAvi05 populations.

At the continental scale, the same sites used in the local adaptation and common-garden studies were compared (CAvi05 and FL06). There was significant differentiation between these sites (genic differentiation exact G test across all loci: P<0.0001) and the estimated pairwise F_st_ value (across all loci) was 0.038.

## Discussion

First, our snapshot surveys on herbivory show that the proportion of fruit damage by the specialist *Utetheisa ornatrix* and the non-specialist polyphagous *Etiella zinckenella* varies among populations even at the regional scale, suggesting that selection pressure on the host may not be homogeneous among populations. Second, our common garden experiments show genetic differences in resistance traits among the host plant populations, suggesting that *C. pallida* populations are genetically differentiated. Several studies have shown that natural populations vary in defensive traits such as resistance to natural enemies [56]–[63]. Indeed, in the best-studied empirical example of plant-herbivore coevolution, Berenbaum, Zangerl and colleagues showed extensive variation in the levels of furanocoumarins in populations of the wild parsnip from different regions and continents [64]–[65]. This variation correlates with the presence and absence, or intensity of attack, of the plant's main herbivore, the parsnip webworm. In our study, the common-garden design indicates that the differences in resistance traits are indeed genetic and not plastic responses. Third, our results show that geographical variation in resistance traits depends on the spatial scale. While some traits differed at the continental scale, others just varied at the regional scale. Our results at the continental scale depend on the population sampled; for example since we did find difference in PA concentration among the three Brazilian populations, the observed similarity in PAs between the Florida and the Brazilian populations would not be the same if we had sampled a different population in Brazil. Our results showing that some traits vary at the regional scale confirm our earlier evidence based on larval performance on fresh fruits that the plant populations are differentiated even at this small scale [18]. These differences may be caused by drift or selection. In other systems, there is evidence that selection trajectories in ecological interactions may diverge among local neighboring populations [16], [66]. Since *C. pallida* is not native to the neotropics, there are two possible explanations for the patterns of variation in resistance reported here. First, it could be the result of rapid evolution; differentiation may have evolved since this plant's introduction (possibly 500 years ago) [19]. Alternatively, it may be the result of multiple introductions of individuals from divergent populations in the native range. In the future, we intend to use molecular markers on *C. pallida* populations from the native and introduced range to discern between these two alternatives.

None of the plant resistance traits studied here completely explains the patterns of adaptation by *U. ornatrix*. First, although PAs are considered the main resistance trait of *Crotalaria* plants [31], [25], in laboratory experiments in which larvae were subjected to artificial diets with 100 times higher concentrations of PAs, we found that performance of *U. ornatrix* is not negatively affect by PAs and sequestration of PAs has no fitness cost [24], this indicates that *U. ornatrix* cannot select for higher PA concentrations in plant populations. In addition, sequestered PAs are used by *U. ornatrix* for mating and defense [22], and laboratory tests show that larvae prefer diets with higher PA concentration (Hoina, Trigo and Cogni unpublished results), suggesting that specialist herbivores may act as natural selection agents that decrease the level of chemical defenses in plant populations [67], and that PAs may be more effective against some non-specialist polyphagous herbivores than against the specialist [24], [68]–[70], as generally occurs in other systems [71], [72]. Therefore, the geographical variation in the concentration of PAs may result from spatial difference in the incidence of specialist and generalist herbivores. Like PAs, EFNs do not explain our laboratory evidence for regional adaptation, although they may affect larvae performance in the field. Nitrogen content varied at the continental scale, the same scale on which adaptation was observed. However, this trait is unlikely to be the main driver trait because larval consumption of seeds from Florida and Brazil did not differ [18]. Finally, trichomes are also unlikely to be the main driver of local adaptation patterns because, in the laboratory tests, leaves were offered only during the first two days, and larval mortality during this period was similar in the two plant populations [18]. Therefore, the patterns of adaptation observed at the continental scale may be driven by another resistance trait not yet measured (possible candidate traits include isoflavonoids, non-protein amino acids and proteinase inhibitors [28]–[32]), or, more likely, by the emerging property of the synergistic effect of several of these traits [27].

Even if we have not identified the traits responsible, our results showing that some plant traits vary at the regional scale, and our previous evidence for plant population differentiation, based on larval performance on unripe seeds [18], indicates that the plant populations are differentiated at the regional scale, and suggests that *U. ornatrix* is subjected to divergent selection. Our evidence of adaptation at the continental scale implies that we can rule out possible constraining factors such as genetic constraints and conflicting selection on the traits related to local adaptation. Why, then, is *U. ornatrix* not adaptively differentiated at the regional scale? The most likely explanation is the unstable population structure of *U. ornatrix*. In our microsatellite study, we showed population differentiation in samples collected in 2005. However, in our samples collected at the same time of the year in 2008 there was no population differentiation, despite the observation of a similar number of alleles per locus (i.e. similar power to detect differentiation), and despite our larger sample size (i.e. larger power to detect differentiation). Even when we just compared the populations sampled in both years, there was differentiation in 2005 and no differentiation in 2008. The lack of differentiation in 2008 indicates high rates of dispersal that can cause ‘genetic swamping’ by replacing locally adapted alleles with locally maladapted alleles common in the metapopulation as whole [17], [73], and can homogenize genetic variation among patches, reducing the supply of novel variation attainable through dispersal [74], [75]. As empirical examples, gene flow reduced adaptation between parsnips and webworms [76], and prevented local adaptation of the scale *Matsucoccus acalyptus* to pinyon pines [77]. The difference in population structure between the years suggests a pattern of local population extinction and recolonization. Indeed, *C. pallida* has a patchy distribution and occurs in habitats where fire and other human disturbances are common, and these can cause local moth extinctions (as occurred in our locality JU). When individual parasite populations are ephemeral, adaptive differentiation may only be found at larger geographical scales [78], [79]. In addition, recolonization may occur by moths originating from populations where alternative host plants are present. Alternative hosts may decrease the level of adaptation to the main host [76]. Our results show no differentiation among populations occurring on the main host, *C. pallida*, and on the alternative host *C. trichotoma*, suggesting the possibility of recolonization of populations from individuals occurring on alternative hosts. In conclusion, temporal change in the herbivore population structure is the most likely cause for the lack of regional adaptation in our system. Our results suggest that sampling through time can enhance understanding of coevolutionary dynamics.

## Supporting Information

Table S1 Microsatellite loci used in the *Utetheisa ornatrix* population structure study.
(DOCX)

Table S2 Test of linkage disequilibrium for pairs of microsatellite loci tested across all populations of *Utetheisa ornatrix*.
(DOCX)

Table S3 Genetic diversity for each *Utetheisa ornatrix* microsatellite locus on each population.
(DOCX)
