# Supplementary material for: Varying Herbivore Population Structure Correlates with Lack of Local Adaptation in a Geographic Variable Plant-Herbivore Interaction
Source: PLoS One. 2011 Dec 29;6(12):e29220. doi: 10.1371/journal.pone.0029220 (PMC3248420; doi:10.1371/journal.pone.0029220)
Supplement: Table S1 — Microsatellite loci used in the Utetheisa ornatrix population structure study. (DOCX) [file pone.0029220.s001.docx]

Table S1. Microsatellite loci used in the *Utetheisa ornatrix* population structure study.

| Locus | Primer sequence (5’-3’) | | No. of alleles | Size range |
| --- | --- | --- | --- | --- |
|  | forward | reverse |  |  |
| Utor2 | TCAACTGTTATTCTTTAAATGTTTG | TCATATCTACGTATAGCTGGTG | 16 | 217-277 |
| Utor7 | TGCTAAGAACGTGTATATTGTAGGAAC | ATATGTGACTCAGAGAAGAAATACAAAG | 10 | 233-273 |
| Utor10 | TCGAGAGCCCCTGTCTGTAAC | CGGGATAAAACATAGCCTATAACC | 7 | 234-274 |
| Utor28 | GGAGAATTGAGGTGCCTCTG | TGGTCACCCATCCATATAATG | 13 | 199-275 |
| UtorTac | GTTTTGCGTGGGTAATTATAA | AGCTGAAGAGTTTGTTTGTTTG | 12 | 198-250 |
