# Supplementary material for: Varying Herbivore Population Structure Correlates with Lack of Local Adaptation in a Geographic Variable Plant-Herbivore Interaction
Source: PLoS One. 2011 Dec 29;6(12):e29220. doi: 10.1371/journal.pone.0029220 (PMC3248420; doi:10.1371/journal.pone.0029220)
Supplement: Table S2 — Test of linkage disequilibrium for pairs of microsatellite loci tested across all populations of Utetheisa ornatrix. (DOCX) [file pone.0029220.s002.docx]

Table S2. Test of linkage disequilibrium for pairs of microsatellite loci tested across all populations of *Utetheisa ornatrix*.

| **Locus pair** | **Χ^2^** | **d.f.** | **p** |
| --- | --- | --- | --- |
| Utor 10 & Utor 28 | 0.00 | 10 | 1.00 |
| Utor10 & Utor 2 | 3.05 | 6 | 0.80 |
| Utor28 & Utor2 | 8.06 | 20 | 0.99 |
| Utor10 & Utor7 | 0.00 | 8 | 1.00 |
| Utor28 & Utor7 | 14.70 | 20 | 0.79 |
| Utor2 & Utor7 | 8.82 | 20 | 0.98 |
| Utor10 & UtorTAC | 0.00 | 8 | 1.00 |
| Utor28 & UtorTAC | 8.14 | 20 | 0.99 |
| Utor2 & UtorTAC | 15.71 | 20 | 0.73 |
| Utor7 & UtorTAC | 18.40 | 20 | 0.56 |
