# Supplementary material for: Varying Herbivore Population Structure Correlates with Lack of Local Adaptation in a Geographic Variable Plant-Herbivore Interaction
Source: PLoS One. 2011 Dec 29;6(12):e29220. doi: 10.1371/journal.pone.0029220 (PMC3248420; doi:10.1371/journal.pone.0029220)
Supplement: Table S3 — Genetic diversity for each Utetheisa ornatrix microsatellite locus on each population. (DOCX) [file pone.0029220.s003.docx]

Table S3. Genetic diversity for each *Utetheisa ornatrix* microsatellite locus on each population.

| Population |  | *Utor2* | Utor7 | Utor10 | Utor28 | *UtorTAC* |
| --- | --- | --- | --- | --- | --- | --- |
| CAvi05 | A | 26 | 28 | 0 | 30 | 30 |
|  | H_e_ | 11.4 | 10.96 | - | 1.93 | 10.28 |
|  | H_o_ | 4* | 12 | - | 2 | 8 |
| CAvi08 | A | 50 | 50 | 16 | 52 | 52 |
|  | H_e_ | 21.39 | 19.26 | 6.20 | 7.37 | 18.39 |
|  | H_o_ | 12* | 19 | 4 | 6 | 12* |
| CAia08 | A | 48 | 50 | 8 | 50 | 50 |
|  | H_e_ | 21.11 | 19.43 | 3 | 11.96 | 19.96 |
|  | H_o_ | 11* | 15 | 2 | 6* | 17 |
| BOvi05 | A | 30 | 30 | 0 | 32 | 32 |
|  | H_e_ | 12.76 | 10.28 | - | 3.61 | 11.97 |
|  | H_o_ | 4* | 7 | - | 4 | 13 |
| BOvi08 | A | 50 | 48 | 12 | 58 | 58 |
|  | H_e_ | 22.65 | 17.87 | 5.09 | 9.12 | 23.95 |
|  | H_o_ | 15* | 16 | 2* | 7 | 20 |
| BOba08 | A | 40 | 46 | 12 | 46 | 46 |
|  | H_e_ | 17.28 | 17.69 | 3.36 | 8.69 | 18.38 |
|  | H_o_ | 10* | 10* | 2 | 6 | 16 |
| JU05 | A | 28 | 32 | 10 | 28 | 24 |
|  | H_e_ | 12.30 | 12.68 | 3.11 | 9.78 | 9.48 |
|  | H_o_ | 4* | 10 | 2 | 5* | 8 |
| LI08 | A | 42 | 48 | 14 | 44 | 48 |
|  | H_e_ | 16.83 | 18 | 5.46 | 7.23 | 18.55 |
|  | H_o_ | 12* | 10* | 1* | 5 | 17 |
| PI08 | A | 38 | 42 | 8 | 44 | 44 |
|  | H_e_ | 17.08 | 16.78 | 2.85 | 7.05 | 16.12 |
|  | H_o_ | 11* | 12 | 0 | 4 | 12 |
| FL06 | A | 24 | 28 | 6 | 30 | 28 |
|  | H_e_ | 10.48 | 10.63 | 2.4 | 2.9 | 11.56 |
|  | H_o_ | 3* | 10 | 0 | 2 | 8 |

Number of alleles (A), expected number of heterozygotes (H_e_) and observed number of heterozygotes (H_o_). * indicates significant deviation from Hardy-Weinberg equilibrium after correction for multiple testing.
